# Implementation of a Rotational Ultrasound Biomicroscopy System Equipped with a High-Frequency Angled Needle Transducer — *Ex Vivo* Ultrasound Imaging of Porcine Ocular Posterior Tissues

**DOI:** 10.3390/s140917807

**Published:** 2014-09-24

**Authors:** Tae-Hoon Bok, Juho Kim, Jinho Bae, Chong Hyun Lee, Dong-Guk Paeng

**Affiliations:** 1 Department of Physics, Ryerson University, 350 Victoria Street, Toronto, ON M5B 0A5, Canada; E-Mail: tbok@ryerson.ca; 2 Department of Ocean System Engineering, Jeju National Univeristy, 102 Jejudaehak-ro, Jeju-si, Jeju-do 690-756, Korea; E-Mails: lizard@jejunu.ac.kr (J.K.); baejh@jejunu.ac.kr (J.B.); chonglee@jejunu.ac.kr (C.H.L.)

**Keywords:** high-frequency ultrasound, rotational scanning, angled needle transducer, automatic motion control

## Abstract

The mechanical scanning of a single element transducer has been mostly utilized for high-frequency ultrasound imaging. However, it requires space for the mechanical motion of the transducer. In this paper, a rotational scanning ultrasound biomicroscopy (UBM) system equipped with a high-frequency angled needle transducer is designed and implemented in order to minimize the space required. It was applied to *ex vivo* ultrasound imaging of porcine posterior ocular tissues through a minimal incision hole of 1 mm in diameter. The retina and sclera for the one eye were visualized in the relative rotating angle range of 270° ∼ 330° and at a distance range of 6 ∼ 7 mm, whereas the tissues of the other eye were observed in relative angle range of 160° ∼ 220° and at a distance range of 7.5 ∼ 9 mm. The layer between retina and sclera seemed to be bent because the distance between the transducer tip and the layer was varied while the transducer was rotated. Certin features of the rotation system such as the optimal scanning angle, step angle and data length need to be improved for ensure higher accuracy and precision. Moreover, the focal length should be considered for the image quality. This implementation represents the first report of a rotational scanning UBM system.

## 1. Introduction

In general, the electrical scanning method with arrayed elements has been mostly developed for diagnostic ultrasound probes. The arrayed probe, however, is usually limited to low frequencies of less than approximately 20 MHz. Recently, high-frequency applications have been highlighted in diagnostic ultrasound for better resolution. Ultrasound biomicroscopy (UBM) systems are frequently used for high**-**frequency applications, and a single element transducer has been mostly utilized with mechanical scanning in high-frequency UBM systems [1]. Moreover, a UBM system with the high-frequency array probes has recently been commercialized [2].

There are several scanning methods to obtain images using a single element transducer. The basic scanning method is a linear scanning resulting in rectangular images [3,4]. An arc scanning method was tried for the concave surface of the eye [5]. A radial scanning is popular for polar images in intravascular ultrasound and endoluminal imaging [6]. A cylindrical C-scan method was tried to get images at a certain depth by combining linear scanning and radial scanning [7]. Finally, a sectorial scanning method was tried in order to determine cyclic variations in blood echogenicity in the human radial artery [8].

Recently a rotational scanning system with an angled needle transducer was proposed for the posterior imaging of the eye [9], since high-frequency ultrasound over 40 MHz is not suitable for the posterior segment due to the high attenuation of ultrasound and thus the limited penetration depth. A 40 MHz angled needle transducer was inserted through a 1 mm hole of the sclera of the porcine eye, and the stage where the target was put on was manually rotated for posterior imaging of the eye. This previous study was performed in order to apply it to effective diagnosis of retina vein occlusion (RVO). The blood flow velocity in the retinal vessel is significantly reduced due to RVO, so that the measurement of retinal blood flow is required for diagnosing RVO, moreover, monitoring of retinal blood flow is essential during eye surgery. Some proposed methods such as the microsphere method [10] and the isotope clearance method [11] were very invasive, so an ultrasound Doppler analysis would be an appropriate proper method to diagnose RVO [12]. For more accurate measurement of variation in blood flow velocity in the retinal vessel, high-frequency (over 40 MHz) ultrasound is required since the diameter of human retinal vessel is less than 200 μm. High-frequency ultrasound of such a range has a higher attenuation. Hence, the needle transducer should be inserted as closely to the posterior segment of the eye as possible with minimal incision. During retinal surgery, surgical instruments are usually inserted into the eye by incising a small hole which is approximately 1 mm in diameter in the sclera. If the needle transducer is inserted into the hall for eye surgery, and the ocular tissue is imaged by the rotational scanning system equipped with the transducer, then the retinal blood flow velocity can be easily obtained, moreover, real-time ocular imaging can be applied to the ultrasound-guided retinal surgery. Prior to rotational scanning, the uses of high-frequency ultrasound needle transducer for characterizing the anterior ocular tissues have been presented in some previous studies [13,14]. These previous studies were focused on the hardness of the cataract lens, so that were not related to B-mode imaging by rotational scanning.

In this paper, an automatic rotational scanning system equipped with a high-frequency angled needle transducer was designed, configured, and implemented for a rotational scanning UBM system. The preliminary *ex vivo* experiments were performed with the porcine eyes to confirm the feasibility of the rotational UBM system. This paper is an extended version of work published in [15]. We extend our previous work by the upgraded system control panel based on a graphic user interface and application of a scan conversion to B-mode image visualization.

## 2. Methods

### 2.1. Animal Experiments

Two porcine eyes of two different pigs were extracted at a local slaughterhouse and kept in formalin. The eyes were fixed at a stand in order to avoid movement. All procedures performed on animals were approved by the Animal Care and Use Committee of Jeju National University in order to ensure that they were appropriate and humane.

### 2.2. Configuration of Rotational Scanning Ultrasound Biomicroscopy

**Figure 1.**
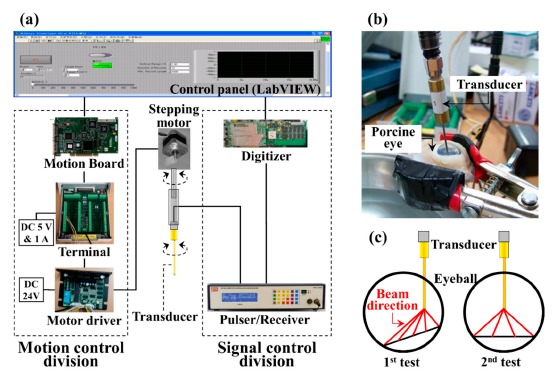
(**a**) Configuration of the rotational scanning ultrasound biomicroscopy system equipped with a high-frequency angled needle transducer. The UBM system was composed of a high-frequency (>40 MHz) angled (5°) needle transducer, and the motion and the signal control divisions; (**b**) Photograph of the needle transducer inserting into a porcine eye; (**c**) A schematic diagram of transducer inserted and the beam direction.

The rotational scanning UBM system was composed of a 40 MHz unfocused lead magnesium niobate-lead titanate (PMN-PT) needle transducer with a 45° angled tip [16,17], and the motion and the signal control divisions as shown in Figure 1. The needle transducer had a rectangular aperture of 0.4 mm by 0.56 mm and a 52% fractional bandwidth [9]. The axial and lateral resolution of the transducer was 56 and 450 μm, respectively. The motion control division consisted of a stepping motor (TS3617-N2E4, Tamagawa, Nagano, Japan), a motor driver (NMD-2336UD, Tamagawa), a terminal (UMI-7764, National Instruments, Austin, TX, USA) and a motion board (PCI-7332, National Instruments). The signal control division comprised a pulser/receiver (5900PR, Olympus Scientific Solutions Americas Inc., Waltham, MA, USA), a digital oscilloscope (LT354, Teledyne LeCroy, Chestnut Ridge, NY, USA) and a high speed digitizer (CompuScope CS122G1, GaGe Applied Technologies, Lockport, NY, USA). The needle transducer was inserted into the porcine eye through a small incision hole of 1 mm diameter for rotational scanning. Ten echo samples from a scan line were collected with 1 kHz of pulse repetition frequency (PRF) at a reference angle of 0° in order to get the averaged signal for the high signal-to-noise ratio. And then the transducer was automatically turned to 0.45° for an increment for the next acquisition process which was controlled by a LabVIEW program. The total steps were 800 for 360°, and the data acquisition time was 10 ms at each step. The system parameters were addressed in Table 1. The acoustic data was transferred to a PC and analyzed in MATLAB R2013a (Mathworks, Natick, MA, USA).

### 2.3. Scan Conversion Algorithm

**Figure 2.**
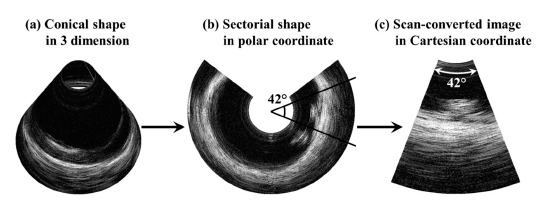
Scan conversion algorithm. (**a**) Acquired image in 3-dimensions by the rotational scanning ultrasound biomicroscopy; (**b**) sectorial image in the polar coordinates obtained by unfolding; (**c**) scan-converted image in Cartesian coordinates.

The scan-conversion algorithm worked as follows and shown in Figure 2: (a) The scanning area of the rotational UBM became a conical shape in 3-dimensions; (b) When the conical shape was unfolded on a 2-dimensional plane, it became a sectorial shape such as a polar coordinate. The scan angle was smaller than the rotational angle 360° of the transducer due to 1/sqrt(2) × 360° = 255°; (c) The polar coordinated image was converted into the Cartesian coordinate by the built-in function ‘pol2cart’ in MATLAB. Hence, the 42° sector image shown in Figure 2c (indicated angle) is the result of mechanical rotation through 60° (1/sqrt(2) × 60° = 42°). The scan-converted image might seem like distortion since a conical shape in 3-dimension is converted into a plane in 2-dimension. However, if the rotational angle of the transducer is small enough, such as less than 10°, then the scanned area which is bent can approximate to a plane, and the scan-converted image seems to be a sector scan image. This would be a potentially expected effect of pseudo-sector scan image on ophthalmic 2-dimensional ultrasound imaging.

## 3. Results and Discussion

### 3.1. LabVIEW Graphic User Interface Based Control Panel for Ultrasound Biomicroscopy

**Figure 3.**
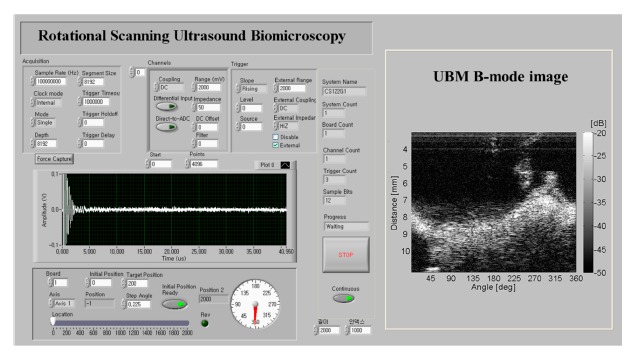
Graphic user interface of the rotational scanning ultrasound biomicroscopy system.

The graphic user interface (GUI) was designed based on LabVIEW in order to operate the rotational scanning UBM, considering the acquired acoustic signals, the transducer rotation controls, and the display of real-time-acquiring signals as shown in Figure 3. The left panel is for the input section which operates the rotational UBM, and the right panel is for the output section which shows the post-processed B-mode image. The upper part of the left panel is for acoustic data acquisition and the data save in a PC, and the lower part controls the rotational motion. While the transducer is rotated, the red needle in the lower part of the left panel is also simultaneously rotated, and the real-time time-domain acoustic signal is shown in the central part of the left panel. This GUI is the first version of the rotational scanning UBM so that it should be improved for the universal control of the UBM. For example, the time-domain acoustic signal is radio-frequency (RF) raw data which is not filtered. For more accurate visualization, the RF signal needs to be filtered by band-pass filter or matched filter. These signal processors should be included into the GUI. In addition, the scanning angle is not adjustable, which means the needle transducer rotates from the reference angle 0° to a certain angle. The reference angle should be adjusted in the upgraded GUI.

### 3.2. B-Mode Posterior Imaging of Porcine Ocular Tissues

**Figure 4.**
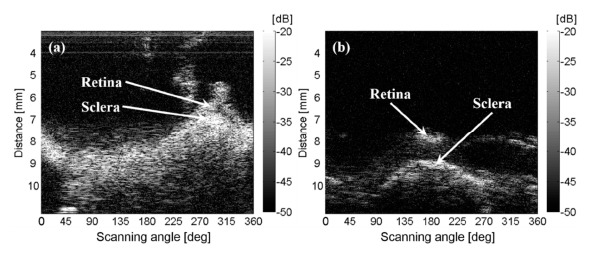
*Ex vivo* B-mode images of porcine ocular posterior tissues by the rotational scanning ultrasound biomicroscopy on the rectangular coordinate. The retina and the sclera were visualized in the relative angle range of 270° ∼ 330° and the distance range of 6 ∼ 7 mm for the 1st eye (**a**); and in the relative angle range of 160° ∼ 220° and the distance range of 7.5 ∼ 9 mm for the 2nd eye (**b**).

**Figure 5.**
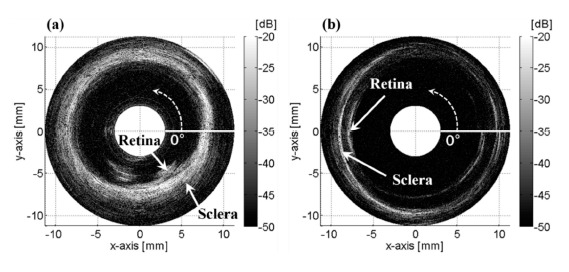
*Ex vivo* B-mode images of porcine ocular posterior tissues by the rotational scanning ultrasound biomicroscopy projected on the polar coordinate. The reference angle 0° was not consistent during one period since the motor could not be accurately controlled. The dotted arrow indicates the scanning direction from the reference angle: (**a**) the 1st eye; (**b**) the 2nd eye.

Figure 4a shows that the retina and the sclera layers in the posterior section of the first porcine eye were visualized in the relative angle range of 270° ∼ 330° and a distance range of 6 ∼ 7 mm in a rectangular B-mode image. The retina and sclera layers were seen as bent layers because the distance from the tip of the angled transducer was changed while rotating. The retina and the sclera for the other eye were obviously visualized in relative angle range of 160° ∼ 220° and at a distance range of 7.5 ∼ 9 mm as shown in Figure 4b. The actual area of tissues cannot be reflected in the rectangular B-mode image because the lateral distance of an arbitrary scanning angle range in the near-field is not consistent with that in the far-field. These rectangular B-mode images were mapped on the polar coordinates in order to verify the continuity in the rotating image as shown in Figure 5. The sclera was not scanned as a circle (Figure 5a) because the transducer inserted was not perpendicular to the surface of the eye (Figure 1c). The distance between the transducer tip and the inner wall of the eye was varied while the obliquely inserted transducer rotated on its axis. The received signal at 0° deviated a little from that at 360° (Figure 5a), and was totally different from that at 360° (Figure 5b). This was because the transducer needle was not aligned with the rotating axis or because the rotating system was inaccurate. There was a little vibrating movement of the motor in every step. This movement resulted in unstable holding and produced a gap or an overlap between each step, so that the beginning (0°) and end (360°) of the reconstructed image were not consistent. Hence, the motor holder should be very stable in order to reduce the motor motion errors. The transducer inserted into the 2nd eye was more perpendicular than that into the 1st eye (Figure 1c), so that the rotated image of the retina and sclera was seen to be the more circular pattern for the 1st measurement than the 2nd one as shown in Figure 4.

**Figure 6.**
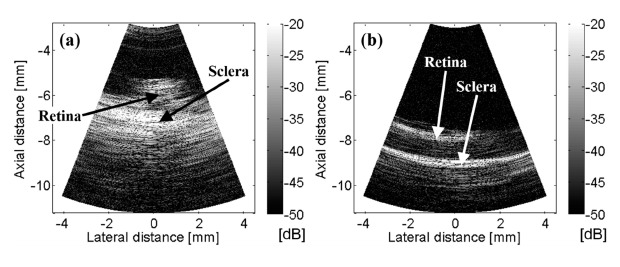
Scan-converted B-mode images of porcine ocular posterior tissues by the rotational scanning ultrasound biomicroscopy. (**a**) Rotating angle of 270° ∼ 330° for the 1st eye; (**b**) rotating angle of 160° ∼ 220° for the 2nd eye.

The rectangular B-mode images in the range of rotating angle 270° ∼ 330° and 160° ∼ 220° were scan-converted as shown in Figure 6, respectively. The scanning area of the rotational UBM is a cone in shape. When a conical shape in 3-dimension is unfolded on the plane, it becomes a sectorial shape in 2-dimension, and the scanning angle is smaller than the rotating angle. Hence, the scanning angular range is 42° for the rotational angular range of 60° which is the region of interest (ROI). The scan-converted image provides a more appropriate size-ratio of the original ROI including width, angle and distance than the B-mode images on the rectangular or polar coordinates, so that it is most useful for the intended application.

### 3.3. Limitation of Study

We confirmed that the rotational scanning UBM equipped with a high-frequency angled needle transducer could be automatically controlled by the motion control system for ultrasound imaging. However, there is room for further development in the areas of the accuracy and the precision of the angle control, and the higher frame rate. Specifically, increased control over the number and size of rotational steps per frame and over the frame rate as well as determination of the scanning angle required for the intended application would enable improved design of future generations of this system. Moreover, the reference angle, 0° should be fixed since the ultrasound image could be shifted if the angle was varied. The pulse repetition frequency (PRF) should be determined considering the frame rate and the number of steps per one frame. The gain also has to be optimized depending on the amplitude of received signals. The acoustic data length of one step should be controlled considering the image processing time. In addition, the Doppler analyzer should be added into the rotational UBM system in order to measure the retinal blood flow velocity [18], and the method to display the scan-converted image in real time should be considered in order to apply to ultrasound-guided eye surgery. The present study represents a preliminary report about imaging ocular tissues with a rotational UBM system. Even though image acquisition, real-time imaging and quantitative results of blood flow were not fully described in this paper, this study should be meaningful as a new ultrasound imaging modality.

## 4. Conclusions

We designed and implemented a rotational scanning UBM system equipped with a high-frequency angled needle transducer, and *ex vivo* images of the retina and sclera of the porcine eyes were obtained by this system. The transducer was automatically rotated by the motion control system with a stepping motor. Simultaneously, the acoustic signal was acquired and post-processed for the B-mode image. The rotational scanning UBM is a novel method to scan with the minimal scanning space through the insertion of the transducer into the biological tissue. Hence, this rotational scanning UBM system can be applied to high resolution imaging of the retina and retinal vein during retinal surgery using a minimally invasive hole. Consequently, this paper suggests a new scanning method of UBM and its applications for high resolution imaging of some internal tissues with a minimal incision which could be utilized as a surgical aid.

## Figures and Tables

**Table 1.** Parameters of the rotational scanning ultrasound biomicroscopy system.

**Parameter**	**Value**
Energy [μJ]	4
Gain [dB]	56
High-pass filter [MHz]	10
Low-pass filter [MHz]	100
Pulse repetition frequency [kHz]	1
Sampling rate [MHz]	100
Vertical resolution [bits]	12
Step angle [°]	0.45
No. of signal per step	10
Total step	800
Duration per step [ms]	10
